# Determinants of COVID-19 vaccine acceptance in Ethiopia: A systematic review and meta-analysis

**DOI:** 10.1371/journal.pone.0269273

**Published:** 2022-06-03

**Authors:** Ayenew Mose, Abebaw Wasie, Solomon Shitu, Kassahun Haile, Abebe Timerga, Tamirat Melis, Tadesse Sahle, Amare Zewdie

**Affiliations:** 1 Department of Midwifery, College of Medicine and Health Science, Wolkite University, Wolkite, Ethiopia; 2 Department of Public Health, College of Medicine and Health Science, Wolkite University, Wolkite, Ethiopia; 3 Department of Medical Laboratory Science, College of Medicine and Health Science, Wolkite University, Wolkite, Ethiopia; 4 Department of Biomedical Science, College of Medicine and Health Science, Wolkite University, Wolkite, Ethiopia; 5 Department of Nursing, College of Medicine and Health Science, Wolkite University, Wolkite, Ethiopia; Seoul National University College of Medicine, REPUBLIC OF KOREA

## Abstract

**Background:**

Vaccination is the promising strategy to control the coronavirus disease 2019 (COVID-19) pandemic. However, the success of this strategy will rely mainly on the rate of vaccine acceptance among the general population. Therefore, this systematic review and meta-analysis aimed to estimate the pooled prevalence of COVID-19 vaccine acceptance and its determinants in Ethiopia.

**Methods:**

We searched PubMed, Scopus, Google Scholar, African Journals Online, and Web of Sciences database to retrieve related articles. Preferred Reporting Items for Systematic Review and Meta-Analysis (PRISMA) guidelines were used for this study. Funnel plot and Eggers test were done to assess publication bias. Cochrane Q-test and I^2^ statistic were done to chick evidence of heterogeneity. Subgroup analysis was computed based on the study region and the study population. Data were extracted using a Microsoft Excel spreadsheet and analyzed using STATA version 14 statistical software. Weighted inverse variance random effect model was run to estimate the pooled prevalence of COVID-19 vaccine acceptance.

**Results:**

A total of 12 studies with 5,029 study participants were included. The pooled prevalence of COVID-19 vaccine acceptance in Ethiopia was 51.64% (95%CI; 43.95%-59.34%). Being male (AOR = 4.46, 1.19–16.77, I^2^ = 88%), having secondary and above educational status (AOR = 3.97, 1.94–8.12, I^2^ = 69%), good knowledge (AOR = 3.36, 1.71–6.61, I^2^ = 93%), and positive attitude (AOR = 5.40, 2.43–12.00, I^2^ = 87%) were determinants of COVID-19 vaccine acceptance in Ethiopia.

**Conclusion:**

The pooled prevalence of COVID-19 vaccine acceptance was low. Being male, having secondary and above educational status, good knowledge, and positive attitude were the determinants of COVID-19 vaccine acceptance. High level of COVID-19 vaccine acceptance among the general population is crucial to achieve herd immunity in the community. Therefore, policymakers, vaccine campaign program planners, and stakeholders should target to improve public awareness of vaccination that enhances vaccine acceptance and in turn helps to control the pandemic.

## Background

The newly emerged coronavirus disease 2019 (COVID-19) is caused by the highly contagious severe acute respiratory syndrome coronavirus 2 (SARS-CoV-2). At the time of its outbreak, no vaccine was available to prevent individuals from contracting the infection [1]. The World Health Organization declared COVID-19 as a global pandemic on March 11, 2020 [2]. Since then, as of 28 March 2022, COVID-19 infects 308,458,509+ confirmed cases, 5,492,595+ deaths globally. Of this, 7,664,386+ confirmed cases and 159,065+ deaths were reported in Africa. Similarly, in Ethiopia COVID-19 infects 446,268+ confirmed cases and 7,042+ deaths [3].

To date, several vaccines are available to combat COVID-19 infection. Vaccination is safe, effective, and it is the most promising strategy to control the COVID-19 pandemic [4]. Enhancing COVID-19 vaccine uptake among the general population is therefore vital for the global community to return to pre-pandemic normalcy. However, vaccine hesitancy has been identified as a major obstacle to achieve a successful vaccination campaign [5]. As of 11 January 2022, a total of 9,194,549,698+ vaccine doses have been administered globally. In Ethiopia, a total of 10,958,395 vaccine doses have been administered [6].

A global systematic review of COVID-19 vaccine acceptance conducted by Shakeel, C.S et al. showed that the vaccine acceptance rates were highest in Ecuador, Malaysia, and Indonesia (>93.3%). However, the lowest acceptance rate was reported in Lebanon (21.0%) [7]. Another systematic review and meta-analysis conducted by Wake A.D in Africa showed that the vaccine acceptance rate was (48.93%) [8]. Evidence revealed that high perceived risk and severity, gender, previous history of influenza vaccination, older age, level of education, and occupation were some of the factors associated with COVID-19 vaccine acceptances [9–12].

Several studies were conducted in Ethiopia to assess the COVID-19 vaccine acceptance and its associated factors [13–15]. However, inconsistencies in findings regarding vaccine acceptance rate and its determinants were reported across different regions of Ethiopia. Moreover, there is no nationally representative pooled data on the prevalence of COVID 19 acceptance in Ethiopia.

Ethiopia has planned to vaccinate 20% of its population by the end of 2021 [16]. Besides, the World Health Organization suggests that every country should vaccinate 40% of the population by the end of 2021 and 70% by mid-2022 [17]. Thus, this systematic review is crucial to understand the overall public acceptance of COVID-19 vaccination and the factors influencing COVID-19 vaccine acceptance. Additionally, the finding will be helpful for governments, policymakers, and stakeholders to formulate strategies that enhance the uptake of vaccines in the respective regions. Therefore, the main objective of this systematic review and meta-analysis was to estimate the pooled prevalence and to identify determinants of COVID-19 vaccine acceptance in Ethiopia.

## Methods

### Study design and setting

We have checked the PROSPERO database (http://www.library.ucsf.edu/) whether published or ongoing projects exist related to the topic to avoid any further duplication. Thus, the finding revealed that there were no ongoing or published articles in the area of this topic. Therefore, the protocol of this review was registered at the PROSPERO database with ID = CRD42022308977. A systematic review and meta-analysis were conducted to estimate the pooled prevalence of COVID-19 vaccine acceptance and its determinants in Ethiopia. Preferred Reporting Items for Systematic Review and Meta-Analysis (PRISMA) guidelines were used for this review [18].

### Search strategies and source of information

Comprehensive literature was searched using international databases such as PubMed, Scopus, Google scholar, African Journals Online, and Web of Sciences to retrieve related articles. Search terms were formulated using PICO guidelines through the online databases. Medical Subject Headings (MeSH) and key terms had been developed using different Boolean operators ‘AND’ and ‘OR’. The following search terms were used: “COVID-19” OR “SARS-CoV-2” AND ‘‘Vaccine” AND “Willingness” OR “Acceptance” OR “Intention” AND “Associated factors” OR “Determinants” OR “Predictors” AND “Ethiopia”.

### Eligibility criteria

The criteria for inclusion includes studies report the prevalence of COVID-19 vaccine acceptance and its determinants. Both published and unpublished including pre-print studies at any time in the English language only were considered. Furthermore, all cross-sectional studies conducted in Ethiopia till the last search day January 7, 2022, were included. Regarding the study period, there was no restriction. Articles without full abstracts or texts and articles reported out of the outcome interest were excluded. Citations without abstracts and/or full-text, commentaries, anonymous reports, letters, editorials, and reviews were excluded after reviewing the full texts.

### Outcome measurements

This study has two main outcomes. The primary outcome was the prevalence of COVID-19 vaccine acceptance. COVID-19 vaccine acceptance is defined as the proportion of participant’s willingness or intention to receive a vaccine (if it once becomes available). Therefore, all included studies were asked the study participants about their intention to receive the COVID-19 vaccine. Thus, if they respond “Yes” to this question they were considered as vaccine acceptance and if they respond “NO” they were considered as vaccine refusal or hesitancy. The secondary outcome was determinants of COVID-19 vaccine acceptance.

### Data extraction

All studies obtained from all databases were exported to Endnote version X8 software to remove duplicate studies. Then after, all studies were exported to Microsoft Excel spreadsheet. Three authors (AW, SS, and KH) independently extracted all the important data using a standardized data extraction form which was adapted from the Joanna Briggs Institute (JBI) data extraction format. For the first outcome (prevalence) the data extraction format included (primary author, study month, study year, year of publication, region, sample size, prevalence (%), study design, study setting, determinant factors (OR, 95%CI), and study quality). Three authors (AT, TS, and AZ) extracted data for the second outcome (associated factors of COVID-19 vaccine acceptance) using 2 by 2 table format. Finally, log odds ratio for each factor was calculated using Review Manager (RevMan) software [19].

### Quality assessment

To assess the quality of each study included in this systematic review and meta-analysis, the modified Newcastle Ottawa Quality Assessment Scale (NOS) for cross-sectional studies was used [20]. Three authors (AW, SS, and KH) assessed the quality of each study (i.e. methodological quality, sample selection, sample size, comparability and the outcome, and statistical analysis of the study). In the case of disagreement between three authors; another three authors (AT, TS, and AZ) involved and resolved the disagreement. All included studies in this systematic review and meta-analysis were cross-sectional.

### Data processing and analysis

Firstly, selected articles were entered into Microsoft Excel spreadsheet format and imported to STATA version 14. statistical software for analysis. Weighted inverse variance random effect model was used to estimate the pooled prevalence of COVID-19 vaccine acceptance in Ethiopia. We used random effect model because there is heterogeneity between studies which was (I^2^> 50%); however, in the case of homogeneous studies, the fixed effect model was used. Cochrane Q-test and I^2^ statistics were computed to assess heterogeneity among all studies. Accordingly, if the result of I^2^ is 0% to 40% it is mild heterogeneity, 30 to 60% would be moderate heterogeneity, 50 to 90% would be substantial heterogeneity; and 75 to 100% would be considerable heterogeneity [21]. Funnel plot and Eggers test were done to assess publication bias [22, 23]. The p value >0.05 indicated that there was no publication bias. Subgroup analysis was done based on the study region and the study population. Forest plot format was used to present the pooled prevalence COVID-19 vaccine acceptance with 95%CI. To identify determinants of COVID-19 vaccine acceptance, Review Manager Software was used.

## Results

### Characteristics of included studies

**Fig 1:**
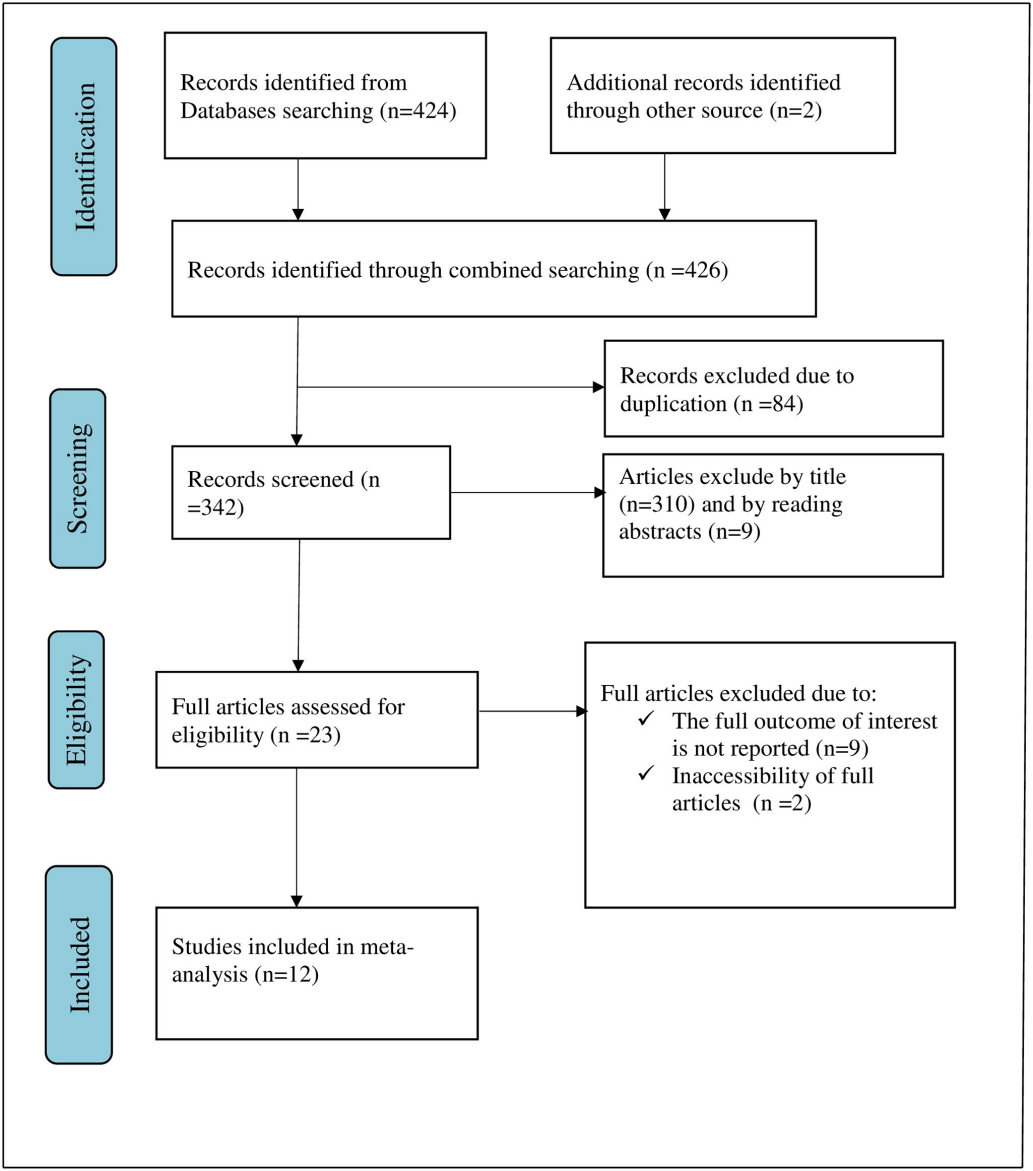
Flow chart of selection for systematic review and meta-analysis on COVID-19 vaccine acceptance and its determinant in Ethiopia, 2021.

Overall, 426 articles were retrieved using a search strategy about COVID-19 vaccine acceptance and associated factors in Ethiopia. Duplicates (84) were removed and 342 articles remained. After reviewing, (n = 310) articles were excluded by title, and (n = 9) articles were excluded by reading abstracts. Therefore, 23 full-text articles were accessed and assessed for inclusion criteria, resulting in further exclusion of 11 articles primarily due to reasons (Fig 1). As a result, 12 studies fulfilled the inclusion criteria to undergo the final systematic review and meta-analysis (Table 1).

**Table 1:** Study characteristics included in the systematic review and meta-analysis on the prevalence of COVID-19 vaccine acceptance and its determinants in Ethiopia.

Authors	Study month	Study year	Year of publication	Region	Sample size	Prevalence (%)	Study design	Study setting	Determinant factors (OR, 95%CI)	Study quality
Angelo AT. et al. [15]	March 15 to 28	2021	2021	SNNP	405	48.4	CS	Facility based	Physicians (9.27, 1.27–27.32), Chronic illness (4.07, 2.02–8.21), Perceived degree of risk of COVID-19 infection (4.63, 1.26–16.98), Positive attitude toward COVID-19 prevention (6.08, 3.39–10.91), Good practices (2.83, 1.58–5.08).	8
Taye BT. et al. [24]	January	2021	2021	Amhara	423	69.3	CS	Institutional based	Good knowledge (2.43, 1.57–3.77), Health science student (2.25, 1.43–3.54), Family practicing COVID-19 prevention (1.73, 1.06–2.81).	7
Abebe H. et al. [25]	March 1 to March 15,	2021	2021	SNNP	492	62.6	CS	Community based	Age ≥46 years (2.36, 1.09–5.39), Secondary and above education (2.59, 1.52–4.39), Chronic disease (3.14, 1.21–8.14), Good knowledge (2.59, 1.67–4.02).	8
Zewude B. & Habtegiorgis T. [26]	March 1 to 30	2021	2021	SNNP	319	46.1	CS	Institutional based	Good attitude (2.83, 1.83–4.37), COVID-19 exists in the study area (0.22, 0.08–0.59), Perception that prevalence and death rate reports of the government are real (0.36, 0.19–0.67), Chronic diseases (2.88, 1.04–7.99), Close relative/friend ever infected by COVID-19 (2.60, 1.12–6.06).	8
Berihun G. et al. [27]	May 1 to 20	2021	2021	Amhara	416	59.4	CS	Institutional based	Health insurance (1.81, 1.70–3.06), Knew anyone diagnosed with COVID-19 (2.48, 1.43–4.32), Good knowledge (6.89, 3.90–120.17), Positive attitude (7.73, 4.02–14.83).	9
Mose A. [28]	February 1 to March 15	2021	2021	SNNP	630	61	CS	Institutional based	Residence (2.5, 1.62–3.91), Educational status (2.8, 1.51–4.21), Immunization counselling (3.4, 1.95–5.91), Good knowledge (2.6, 1.84–3.47), Good adherence to COVID-19 mitigation measures (3.2, 1.91–5.63).	8
Shitu K. et al. [29]	December 2020 to February 2021	2020–2021	2021	Amhara	301	40.8	CS	Institutional based	Male (3.23, 1.70–6.14), Private school teacher (3.27, 1.76–6.10), Perceived susceptibility to COVID-19 (2.69, 1.38–5.24), Perceived seriousness of COVID-19 (4.04,1.80–9.1), Perceived benefit of COVID-19 vaccine (3.0, 1.41–6.34).	9
Hailemariam S. et al. [13]	February 1 to March 1	2021	2021	SNNP	412	31.3	CS	Facility based	Secondary school and above (4.24, 2.23–9.32), Residing in urban areas (2.57, 1.22–5.40), Being compliant with coronavirus disease 2019 guidelines (5.86,3.40–10.09), Good perception toward coronavirus disease 2019 vaccine (3.04, 1.64–5.62).	9
Mesfin Y. et al. [30]	March 1 to April 28	2021	2021	SNNP	398	33.7	CS	Facility based	Chronic diseases (2, 1.08–3.44), Male sex (5, 2.96–8.68), Good knowledge of COVID-19 practice (4.1 2.33–7.31).	8
Mesele M. [14]	April 1 to 30	2021	2021	SNNP	415	45.5	CS	Community based	Sex (2.15, 1.29–3.56), Educational status (3.09, 1.50–6.37), Mass media (1.97, 1.06–3.63), Received any vaccination (5.16, 2.44–10.92), Family members diagnosed with COVID-19 (4.40, 2.1–9.25), Friends diagnosed with COVID-19 (3.91, 1.52–10.04), Tested for COVID-19 (4.40, 1.70–11.36).	7
Mose A. & Yeshaneh A. [31]	January 1 to 30	2021	2021	SNNP	396	70.7	CS	Institutional based	Maternal age (34–41) years (1.46, 1.22–5.13), Educational status (3.48, 1.52–7.95), Good knowledge (5.95, 3.15–7.07), Good practice (9.15, 8.73–12.19).	9
Admasu FT. [32]	May to August	2021	2021	Addis Ababa	422	36.02	CS	Institutional based	Younger age (2.73, 0.18, 4.51), Female (6.4,0.7–13.8), having information about COVID-19 vaccine (6.9, 3.1–15.2), COVID-19 infection history (6.0, 2.5–11.8), Duration since cancer diagnosis (≥10 years) (6.2, 2.6–14.7), Belief about the likelihood of dying of COVID-19 infection (3.05, 1.03–4.05).	8

NB. CS; Cross-sectional, SNNP; Southern Nation Nationalists and People

### COVID-19 vaccine acceptance in Ethiopia

**Fig 2:**
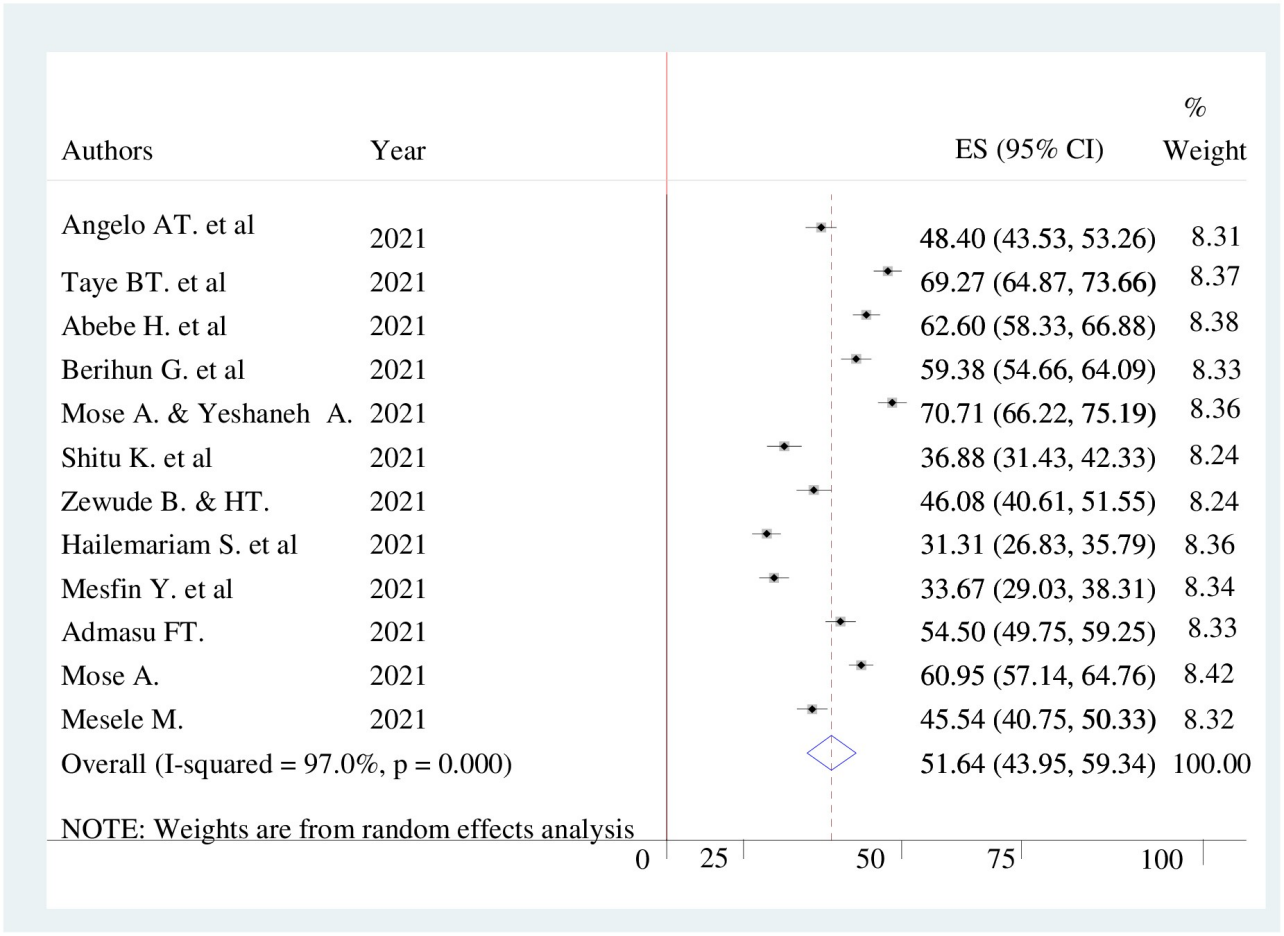
Pooled prevalence of COVID-19 vaccine acceptance in Ethiopia, 2021.

The pooled prevalence of COVID-19 vaccine acceptance in Ethiopia was 51.64% (95%CI; 43.95%-59.34%), with the Cochrane heterogeneity index (I^2^ = 97%), P = 0.000, showing substantial heterogeneity of different studies (I^2^>50%). The finding was presented using a forest plot (Fig 2).

### Publication bias

**Fig 3:**
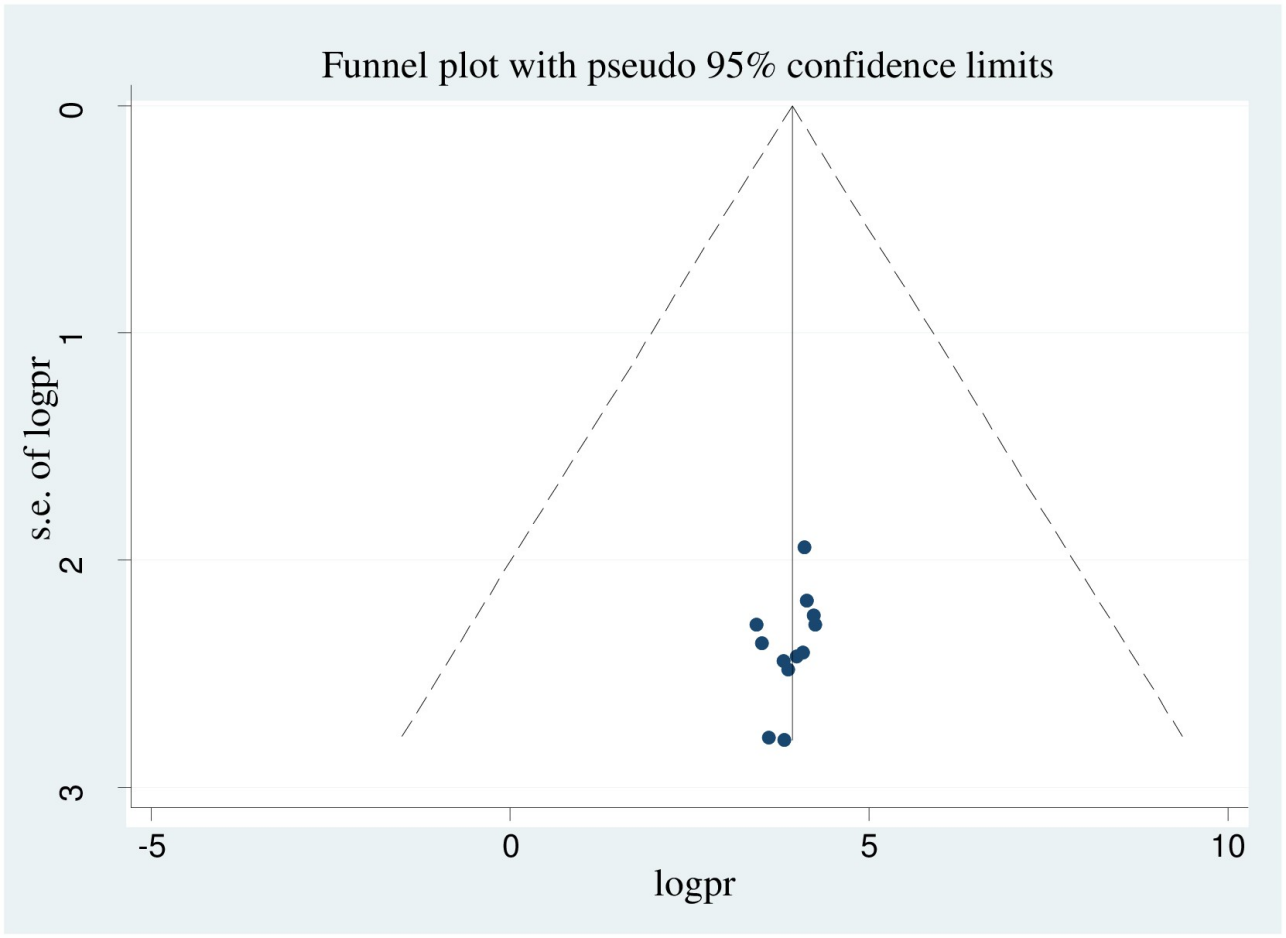
Funnel plot showing symmetric distribution of articles on COVID-19 vaccine acceptance in Ethiopia, 2021.

In this systematic review and meta-analysis, a funnel plot was done to check the presence of publication bias at a significance level of less than 0.05. The Egger’s regression test was not statistically significant (p>0.05) confirming no evidence of publication bias, as presented by the funnel plot (Fig 3).

### Subgroup analysis of COVID-19 vaccine acceptance

**Fig 4:**
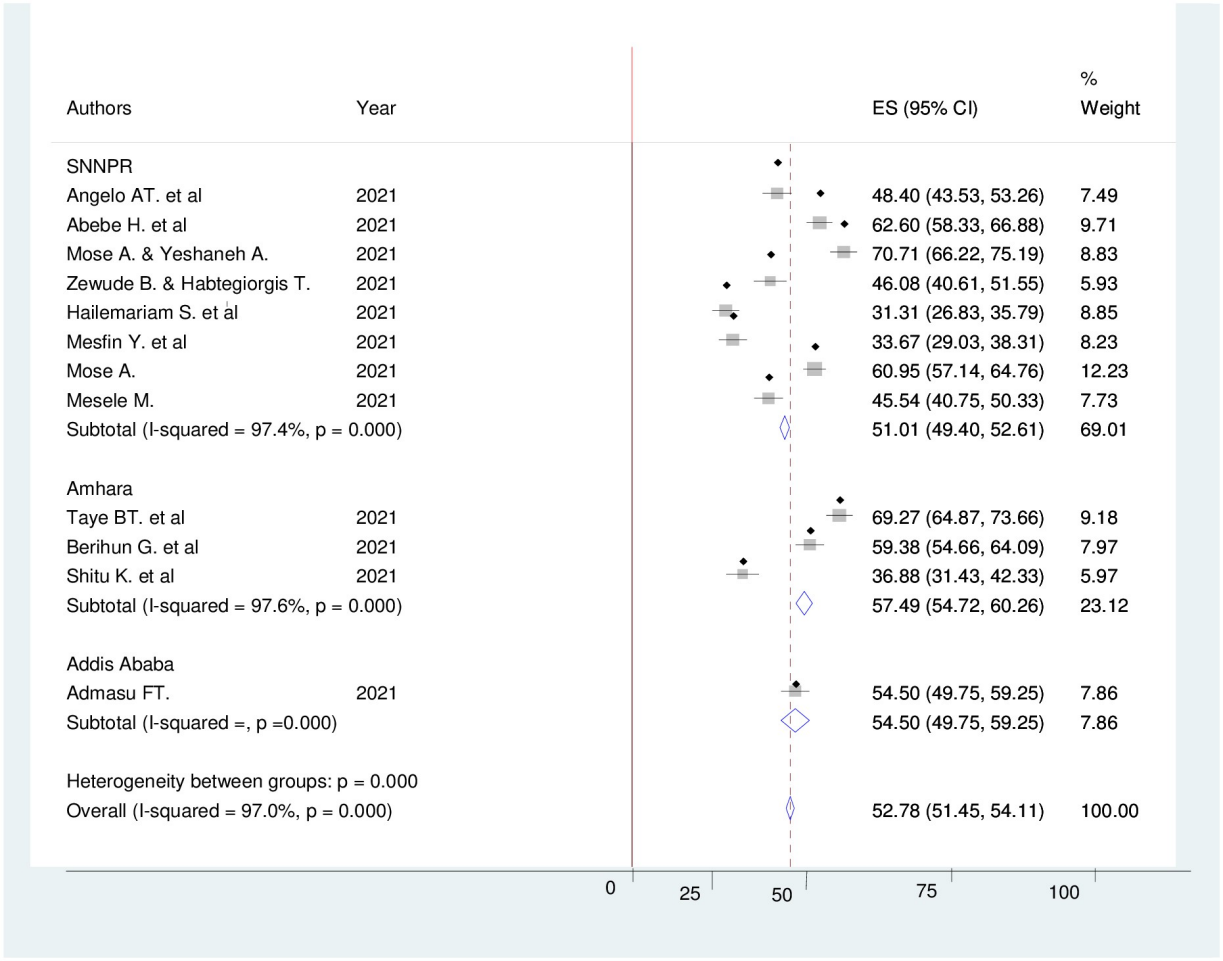
Forest plot showing sub group analysis of COVID-19 vaccine acceptance and its determinant factors by region in Ethiopia, 2021.

**Fig 5:**
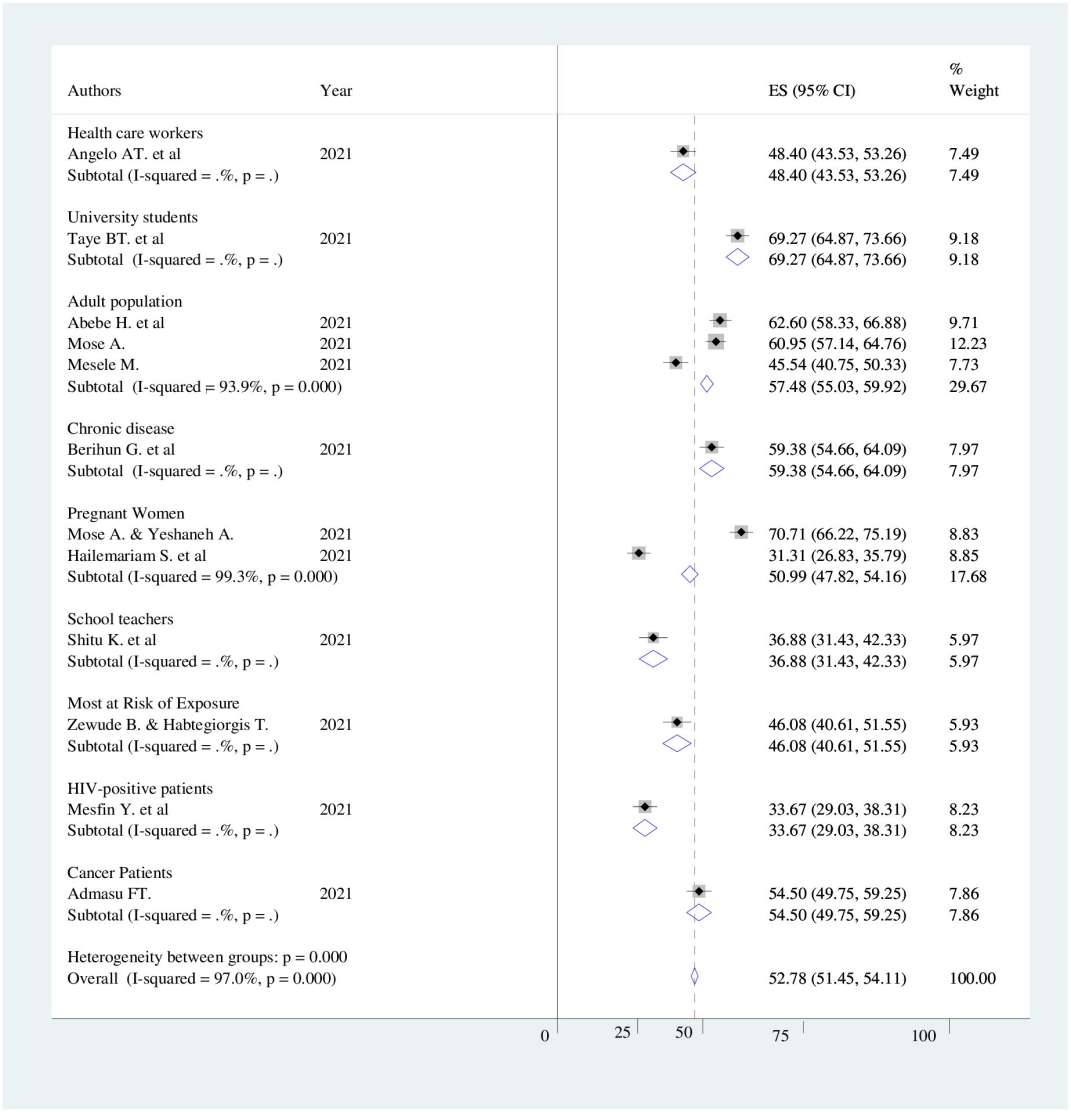
Forest plot showing sub group analysis of COVID-19 vaccine acceptance and its determinant factors by study population in Ethiopia, 2021.

The finding of subgroup analysis by the study region showed that the pooled prevalence of COVID-19 vaccine acceptance was highest in the Amhara region (57.49% (95% CI: 54.72–60.26), I^2^ = 97.6%, p <0.001) and lowest in the Southern Nation Nationalists and People (SNNP) region (51.01% (95% CI: 49.40–52.61), I^2^ = 97.4%, p <0.001) (Fig 4). Moreover, the subgroup analysis by the study population showed that the highest COVID-19 vaccine acceptance was reported among university students 69.27% (95%CI: 64.87–73.66) (Fig 5).

### Sensitivity analysis

**Fig 6:**
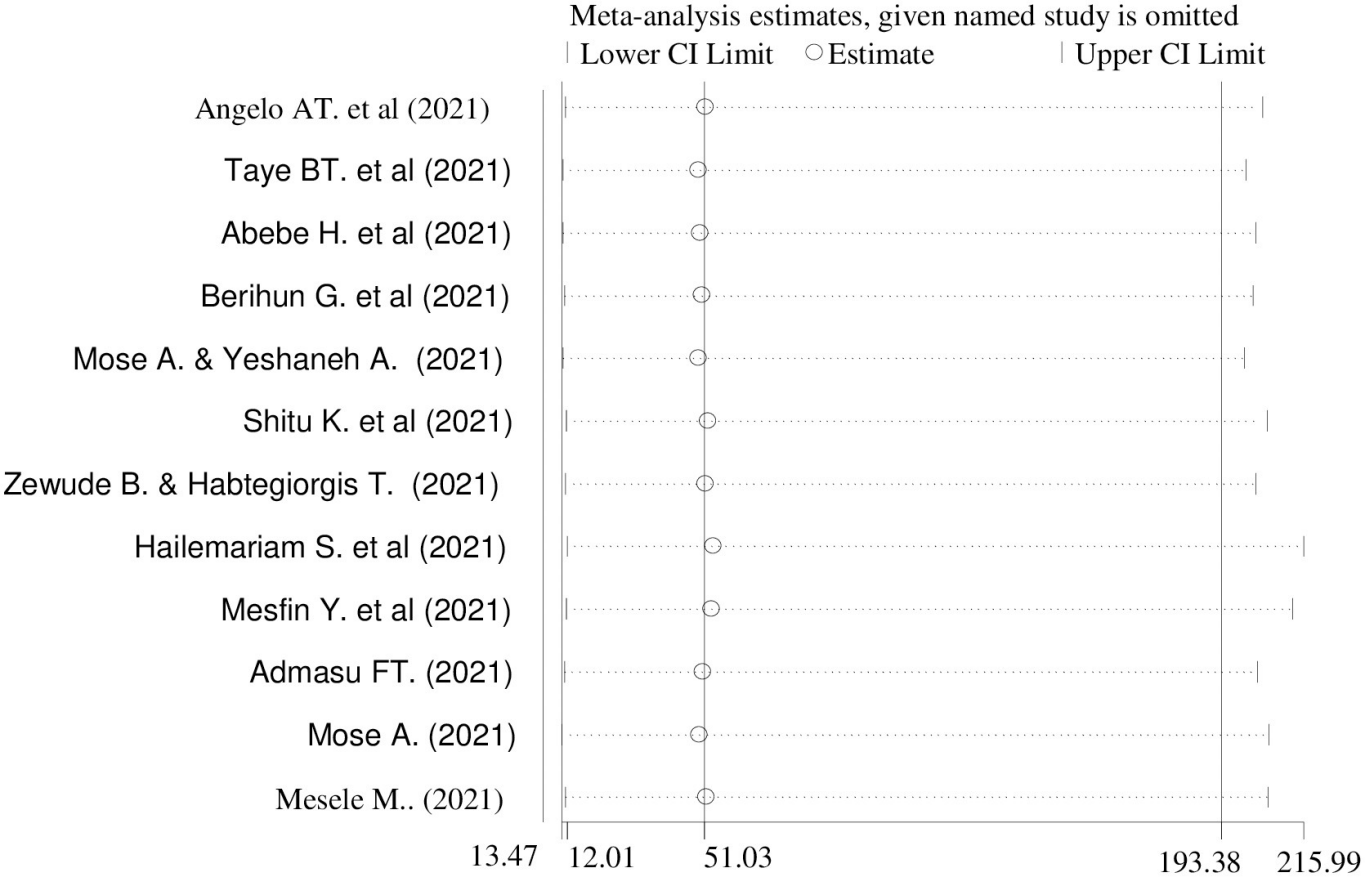
Sensitivity analysis of the prevalence of COVID-19 vaccine acceptance and its determinant factors in Ethiopia, 2021.

A random effect model result showed that no single study has influenced the overall pooled prevalence of COVID-19 vaccine acceptance in Ethiopia (Fig 6).

### Determinants of COVID-19 vaccine acceptance

In this systematic review and meta-analysis; being male, having secondary and above educational status, good knowledge, and positive attitude were determinates of COVID-19 vaccine acceptance in Ethiopia.

The odds of being male were 4.46 times more likely to accept the COVID-19 vaccine than female (AOR = 4.46, 1.19–16.77, I^2^ = 88%). The odds of having secondary and above educational status were 3.97 times more likely to accept the COVID-19 vaccine compared to those who did not have formal education (AOR = 3.97, 1.94–8.12, I^2^ = 69%). The odds of having good knowledge towards the COVID-19 vaccine were 3.36 times more likely to accept the COVID-19 vaccine than those who had poor knowledge (AOR = 3.36, 1.71–6.61, I^2^ = 93%). The odds of having a positive attitude towards the COVID-19 vaccine were 5.40 times more likely to accept the COVID-19 vaccine than those who had poor attitude (AOR = 5.40, 2.43–12.00, I^2^ = 87%).

## Discussion

COVID-19 vaccination is one of the most promising strategies to end the pandemic. However, vaccine hesitancy among the general population could be a massive barrier to get rid of the morbidity and mortality caused by COVID-19 infection [33, 34]. The present systematic review and meta-analysis found that the pooled prevalence of COVID-19 vaccine acceptance in Ethiopia was 51.64%. Being male, having secondary and above educational status, good knowledge, and positive attitude towards COVID-19 vaccine were determinants of COVID-19 vaccine acceptance.

In this study, the pooled prevalence (51.64%) is in line with systematic reviews conducted in Africa (48.93%), Kuwait (53.1%), and Somalia (59.4%) [8, 35, 36]. However, the finding is lower than other systematic reviews conducted at the global level (71.5%), India (86.3%), Thailand (95.7%), and Bangladesh (65.5%) [37–40]. Likewise, the pooled prevalence is higher than studies conducted in Nigeria (40%) and Tunisia (35%) [41, 42]. The possible explanation for varieties of findings might be due to the study period and study population difference. For instance, a study conducted in Thailand was among a physician who works at a university teaching hospital that might have good awareness about the merits of vaccination against infectious diseases compared to the general population.

The subgroup analysis between regions indicated that the highest prevalence of willingness to receive the COVID-19 vaccine was reported in the Amhara region (57.49%). The finding is much lower than studies conducted in China (90%) and Japan (61%) [43, 44]. However, the finding is higher than a study conducted in Jordan (37.4%) [10]. The possible explanation for the difference might be due to the study period difference. For example, studies in China and Japan were conducted during the early phase of the COVID-19 outbreak when the vaccine was not available and even there were no conspiracy theories regarding the effectiveness and side effects of vaccination at the time. Thus, during the early phase of the COVID-19 outbreak people might have more intention to receive COVID-19 vaccine.

The finding of the meta-analysis shows that the odds of being male were 4.46 times more likely to accept COVID-19 vaccine than female. The result is in line with a study conducted in the United States that showed being male were 72% more likely to accept the COVID-19 vaccine compared to female [45]. The possible explanation might be due to differences in decision-making and perceived risk between men and women. The finding is supported by a study conducted by Weber et al. stated that men are more likely to take health/safety risks than women [46].

The odds of having good knowledge towards COVID-19 vaccine were 3.36 times more likely to accept COVID-19 vaccine than those who had poor knowledge. The finding is consistent with studies conducted in China, Palestine, and Somalia [36, 47, 48]. The possible explanation could be those who had awareness of the vaccine might know the benefit of being vaccinated such as halting the transmission of new COVID-19 infections and preventing the possibility of further morbidity and mortality caused by infections.

The willingness to accept the COVID-19 vaccine among those who have secondary and above educational status were 3.97 times more likely than those who did not have formal education. The finding is consistent with a study conducted in China [49]. This could be explained that education enables people to read about the safety of the available vaccine on different websites, magazines, and newspapers. In cumulative, they will know more about the merits of being vaccinated and they might have an intention to receive the available vaccine compared to their counterparts.

The odds of having a positive attitude towards the COVID-19 vaccine were 5.4 times more likely to accept the COVID-19 vaccine than those who had a poor attitude. The finding is similar with previous studies conducted in Thailand and South Carolina [37, 50]. The possible explanation might be having a positive attitude towards vaccination and its potential for prevention of further complications associated with COVID-19 might make people show a willingness to receive the available COVID-19 vaccine.

Overall, the finding revealed that there was low uptake of the COVID-19 vaccine among the general population in Ethiopia compared to other systematic reviews and meta-analyses [51]. To this end, the health care workers and stakeholders should provide outreach health education and should enhance public knowledge about the COVID-19 vaccine. Governments and policymakers should use mass media to provide up-to-date COVID-19 vaccine information and in turn, helps to increase the acceptance of the COVID-19 vaccine in the general population. Moreover, further studies will be needed to address the remaining regions of Ethiopia where vaccine acceptance is not studied such as the Oromiya, Tigray, Somali, and Afar regions.

### Strength and limitation of study

The main strength of this study was we searched five databases to retrieve related articles and we strictly follow PRISMA flow charts. However, this study is not void of limitations. Firstly, web searches were restricted only to the English language. Secondly, the absence of studies in some regions of Ethiopia (i.e. Oromiya, Tigray, Somali, and Afar region) might raise questions regarding the generalizability of the finding. Thirdly, at the time of database searches, there were barriers to the accessibility of the internet to complete an online survey. Moreover, all studies were published in the same year (2021) which might make it unable to see the trends of COVID-19 vaccine acceptance by year.

## Conclusion

The pooled prevalence of COVID-19 vaccine acceptance was low. Nearly one in two adults is vaccine-hesitant in Ethiopia. Gender, educational status, good knowledge, and positive attitude were the determinants of COVID-19 vaccine acceptance. High level of COVID-19 vaccine acceptance among the general population is crucial to achieve herd immunity in the community. Therefore, policymakers and stakeholders should target to improve public awareness and in turn enhance COVID-19 vaccine acceptance to control the pandemic. Furthermore, research is needed to identify determinants of vaccine acceptance in different regions of Ethiopia.

## Supporting information

S1 File Preferred Reporting Items for Systematic Reviews and Meta-Analyses (PRISMA) Guideline. (DOCX)

S2 File Newcastle-Ottawa Quality Assessment Scale of 12 cross sectional studies included in the review of the status of COVID-19 vaccine acceptance and its determinants in Ethiopia. (DOCX)

## Abbreviations

COVID-19: Coronavirus Disease 2019
PRISMA: Preferred Reporting Items for Systematic Reviews and Meta-Analyses
SARS-COV-2: Severe Acute Respiratory Syndrome Coronavirus 2
WHO: World Health Organization

## References

[1] Baloch S, Baloch MA, Zheng T, Pei X. The coronavirus disease 2019 (COVID-19) pandemic. Tohoku J Exp Med. 2020;250(4):271–8. doi: 10.1620/tjem.250.271 32321874

[2] Cucinotta D, Vanelli M. WHO declares COVID-19 a pandemic. Acta Biomed. 2020;91(1):157–60. doi: 10.23750/abm.v91i1.9397 32191675 PMC7569573

[3] World Health Organization W. WHO Coronavirus (COVID-19) Dashboard [Internet]. [cited 1BC Mar 29]. https://covid19.who.int/region/afro/country/et

[4] Tavilani A, Abbasi E, Kian Ara F, Darini A, Asefy Z. COVID-19 vaccines: Current evidence and considerations. Metab Open [Internet]. 2021;12(September):100124. doi: 10.1016/j.metop.2021.100124 34541483 PMC8433053

[5] Dhama K, Sharun K, Tiwari R, Dhawan M, Bin Emran T, Rabaan AA, et al. COVID-19 vaccine hesitancy—reasons and solutions to achieve a successful global vaccination campaign to tackle the ongoing pandemic. Hum Vaccin Immunother [Internet]. 2021;17(10):3495–9. Available from: doi: 10.1080/21645515.2021.1926183 34191680 PMC8437517

[6] World Health Organization W. WHO Coronavirus (COVID-19) Dashboard [Internet]. [cited 2022 Jan 12]. https://covid19.who.int/?ref=vc.ru

[7] Shakeel CS, Mujeeb AA, Mirza MS, Chaudhry B. Global COVID-19 Vaccine Acceptance: A Systematic Review of Associated Social and Behavioral Factors. 2022; 10.3390/vaccines10010110 PMC8779795 35062771

[8] Wake AD. The Acceptance Rate Toward COVID-19 Vaccine in Africa: A Systematic Review and Meta-analysis. Glob Pediatr Heal. 2021;8. doi: 10.1177/2333794X211048738 34616860 PMC8488505

[9] Al-Qerem WA, Jarab AS. COVID-19 Vaccination Acceptance and Its Associated Factors Among a Middle Eastern Population. Front Public Heal. 2021;9(February):1–11. doi: 10.3389/fpubh.2021.632914 33643995 PMC7902782

[10] El-Elimat T, AbuAlSamen MM, Almomani BA, Al-Sawalha NA, Alali FQ. Acceptance and attitudes toward COVID-19 vaccines: A cross-sectional study from Jordan. PLoS One [Internet]. 2021;16(4 April):1–15. Available from: doi: 10.1371/journal.pone.0250555 33891660 PMC8064595

[11] Joshi A, Kaur M, Kaur R, Grover A, Nash D, El-Mohandes A. Predictors of COVID-19 Vaccine Acceptance, Intention, and Hesitancy: A Scoping Review. Front Public Heal. 2021;9(August). 10.3389/fpubh.2021.698111 PMC8414566 34485229

[12] Lau JFW, Woon YL, Leong CT, Teh HS. Factors influencing acceptance of the COVID-19 vaccine in Malaysia: a web-based survey. Osong Public Heal Res Perspect. 2021;12(6):361–73. doi: 10.24171/j.phrp.2021.0085 34818501 PMC8721269

[13] Hailemariam S, Mekonnen B, Shifera N, Endalkachew B, Asnake M, Assefa A, et al. Predictors of pregnant women’s intention to vaccinate against coronavirus disease 2019: A facility-based cross-sectional study in southwest Ethiopia. SAGE Open Med. 2021;9:205031212110384. doi: 10.1177/20503121211038454 34434555 PMC8381422

[14] Mesele M. COVID-19 Vaccination Acceptance and Its Associated Factors in Sodo Town, Wolaita Zone, Southern Ethiopia: Cross-Sectional Study. 2021;2361–7. 10.2147/IDR.S320771 PMC8238544 34194232

[15] Angelo AT, Alemayehu DS, Dachew AM. Health care workers intention to accept COVID-19 vaccine and associated factors in southwestern Ethiopia, 2021. PloS one. 2021 Sep 3;16(9):e0257109. doi: 10.1371/journal.pone.0257109 34478470 PMC8415602

[16] Yazew BG, Abate HK, Mekonnen CK. Knowledge, attitude and practice towards covid-19 in ethiopia: A systematic review; 2020. Patient Prefer Adherence. 2021;15:337–48. doi: 10.2147/PPA.S288186 33623375 PMC7894797

[17] World Health Organization W. WHO, UN set out steps to meet world COVID vaccination targets [Internet]. [cited 2003 Feb 20]. https://www.who.int/news/item/07-10-2021-who-un-set-out-steps-to-meet-world-covid-vaccination-targets

[18] PRISMA TRANSPARENT REPORTING of SYSTEMATIC REVIEWS and META-ANALYSES [Internet]. [cited 2021 Nov 18]. http://www.prisma-statement.org/PRISMAStatement/Checklist.aspx

[19] Review Manager software (RevMan version 5.4.1) [Internet]. https://medium.com/mlearning-ai/review-manager-software-revman-version-5-4-1-933c3d9f7a2f

[20] GA Wells, B Shea, D O’Connell, J Peterson, V Welch, M Losos, et al. The Newcastle-Ottawa Scale (NOS) for assessing the quality of nonrandomised studies in meta-analyses [Internet]. [cited 1BC Feb 1]. http://www.ohri.ca/programs/clinical_epidemiology/oxford.asp

[21] Norhayati MN, Che Yusof R, Azman YM. Systematic Review and Meta-Analysis of COVID-19 Vaccination Acceptance. Front Med. 2022;8(January):1–13. doi: 10.3389/fmed.2021.783982 35155467 PMC8828741

[22] Sterne JA EM. Funnel plots for detecting bias in meta-analysis: guidelines on choice of axis. J Clin Epidemiol. 54(2):1046–55. doi: 10.1016/s0895-4356(01)00377-8 11576817

[23] Sterne JA E M. Regression methods to detect publication and other bias in meta-analysis. Publication bias in meta-analysis: Prevention, assessment and adjustments.: 99–110.

[24] Taye BT, Amogne FK, Demisse TL, Zerihun MS, Kitaw TM, Tiguh AE, et al. Coronavirus disease 2019 vaccine acceptance and perceived barriers among university students in northeast Ethiopia: A cross-sectional study. Clin Epidemiol Glob Heal [Internet]. 2021;12(August):100848. Available from: doi: 10.1016/j.cegh.2021.100848 34395948 PMC8351076

[25] Abebe H, Shitu S, Mose A. Understanding of COVID-19 vaccine knowledge, attitude, acceptance, and determinates of COVID-19 vaccine acceptance among adult population in Ethiopia. Infect Drug Resist. 2021;14:2015–25. doi: 10.2147/IDR.S312116 34103948 PMC8179743

[26] Zewude B, Habtegiorgis T. Willingness to Take COVID-19 Vaccine Among People Most at Risk of Exposure in Southern Ethiopia. Pragmatic Obs Res. 2021;Volume 12:37–47. doi: 10.2147/POR.S313991 34079423 PMC8166351

[27] Berihun G, Walle Z, Berhanu L, Teshome D. Acceptance of covid-19 vaccine and determinant factors among patients with chronic disease visiting dessie comprehensive specialized hospital, northeastern ethiopia. Patient Prefer Adherence. 2021;15:1795–805. doi: 10.2147/PPA.S324564 34429591 PMC8380286

[28] Mose A. Willingness to Receive COVID-19 Vaccine and Its Determinant Factors Among Lactating Mothers in Ethiopia: A Cross-Sectional Study. 2021;4249–59. 10.2147/IDR.S336486 PMC8523808 34703251

[29] Shitu K, Wolde M, Handebo S, Kassie A. Correction to: Acceptance and willingness to pay for COVID-19 vaccine among school teachers in Gondar City, Northwest Ethiopia (Tropical Medicine and Health, (2021), 49, 1, (63), 10.1186/s41182-021-00337-9). Trop Med Health. 2021;49(1). doi: 10.1186/s41182-021-00337-9 34372943 PMC8352142

[30] Mesfin Y. Factors Associated with Intention to Receive COVID-19 Vaccine Among HIV Positive Patients Attending ART Clinic in Southwest Ethiopia. 2021;2731–8. 10.2147/PPA.S342801 PMC8668249 34916783

[31] Mose A, Yeshaneh A. COVID-19 vaccine acceptance and its associated factors among pregnant women attending antenatal care clinic in southwest ethiopia: Institutional-based cross-sectional study. Int J Gen Med. 2021;14:2385–95. doi: 10.2147/IJGM.S314346 34135622 PMC8197585

[32] Admasu FT. Knowledge and proportion of covid-19 vaccination and associated factors among cancer patients attending public hospitals of addis ababa, ethiopia, 2021: A multicenter study. Infect Drug Resist. 2021;14(September):4865–76. 34848979 10.2147/IDR.S340324 PMC8627267

[33] Dror AA, Eisenbach N, Taiber S, Morozov NG, Mizrachi M, Zigron A, et al. Vaccine hesitancy: the next challenge in the fight against COVID-19. Eur J Epidemiol [Internet]. 2020;35(8):775–9. Available from: doi: 10.1007/s10654-020-00671-y 32785815 PMC8851308

[34] Mose A, Haile K, Timerga A. COVID-19 vaccine hesitancy among medical and health science students attending Wolkite University in Ethiopia. PLoS One [Internet]. 2022;17(1 1):1–17. Available from: doi: 10.1371/journal.pone.0263081 35077504 PMC8789154

[35] Kukreti S, Rifai A, Lin C. Willingness to obtain COVID-19 vaccination in general population: A systematic review and meta-analysis. 2022;12.

[36] Ahmed AY, Ahmed MY, Saeed FA, Saeed FA. Level of Acceptance of COVID-19 Vaccine and Its Determinants among High Risk Groups for Severe COVID-19 Infection Living in Mogadishu Somalia. Health (Irvine Calif). 2021;13(11):1206–21.

[37] Sirikalyanpaiboon M, Ousirimaneechai K, Phannajit J, Pitisuttithum P, Jantarabenjakul W, Chaiteerakij R, et al. COVID-19 vaccine acceptance, hesitancy, and determinants among physicians in a university-based teaching hospital in Thailand. BMC Infect Dis [Internet]. 2021;21(1):1–12. Available from: doi: 10.1186/s12879-021-06863-5 34809607 PMC8607407

[38] Sharun K, Faslu Rahman CK, Haritha C V., Jose B, Tiwari R, Dhama K. Covid-19 vaccine acceptance: Beliefs and barriers associated with vaccination among the general population in india. J Exp Biol Agric Sci. 2020;8(Special Issue 1):S210–S.

[39] Lazarus J V., Ratzan SC, Palayew A, Gostin LO, Larson HJ, Rabin K, et al. A global survey of potential acceptance of a COVID-19 vaccine. Nat Med [Internet]. 2021;27(2):225–8. Available from: doi: 10.1038/s41591-020-1124-9 33082575 PMC7573523

[40] Alqudeimat Y, Alenezi D, Alhajri B, Alfouzan H, Almokhaizeem Z, Altamimi S, et al. Acceptance of a COVID-19 vaccine and its related determinants among the general adult population in Kuwait. Med Princ Pract. 2021;30(3):262–71. doi: 10.1159/000514636 33486492 PMC8089409

[41] Mustapha M, Kankia, Basira L, Sha A, Jatau I, Wada AS, et al. Factors associated with acceptance of COVID- 19 vaccine among University health sciences students in Northwest Nigeria. 2021;1–15. Available from: doi: 10.1371/journal.pone.0260672 34843594 PMC8629299

[42] Khiari H, Cherif I, Fehmi M, Mezlini A. COVID-19 Vaccination Acceptance and Its Associated Factors among Cancer Patients in Tunisia. 2021;22:3499–506. 10.31557/APJCP.2021.22.11.3499 PMC9068194 34837905

[43] Wang J, Jing R, Lai X, Zhang H, Lyu Y, Knoll MD, et al. Acceptance of covid-19 vaccination during the covid-19 pandemic in china. Vaccines. 2020;8(3):1–14. doi: 10.3390/vaccines8030482 32867224 PMC7565574

[44] Machida M, Nakamura I, Kojima T, Saito R, Nakaya T, Hanibuchi T, et al. Acceptance of a covid-19 vaccine in japan during the covid-19 pandemic. Vaccines. 2021;9(3):1–11. doi: 10.3390/vaccines9030210 33802285 PMC8002097

[45] Kelly BJ, Southwell BG, McCormack LA, Bann CM, MacDonald PDM, Frasier AM, et al. Correction to: Predictors of willingness to get a COVID-19 vaccine in the U.S (BMC Infectious Diseases, (2021), 21, 1, (338), 10.1186/s12879-021-06023-9). BMC Infect Dis. 2021;21(1):1–7.33390160

[46] Weber Elke U., Ann-Renée Blais NEB. A domain-specific risk-attitude scale: measuring risk perceptions and risk behaviors. J Behav Decis Mak. 2002;15(4):263–90.

[47] Maraqa B, Nazzal Z, Rabi R, Sarhan N, Al-shakhra K. COVID-19 vaccine hesitancy among health care workers in Palestine: A call for action. Diabetes Metab Syndr. 2020;14(4)(January):337–9. 33992654 10.1016/j.ypmed.2021.106618 PMC8117476

[48] Tao L, Wang R, Han N, Liu J, Yuan C, Deng L, et al. Acceptance of a COVID-19 vaccine and associated factors among pregnant women in China: a multi-center cross-sectional study based on health belief model. Hum Vaccines Immunother [Internet]. 2021;17(8):2378–88. Available from: doi: 10.1080/21645515.2021.1892432 33989109 PMC8475603

[49] Pan J, Kezhong A, Liu Z, Zhang P, Xu Z, Guo X, et al. Factors That Impact Acceptance of COVID-19 Vaccination in Different Community-Dwelling Populations in China. Vaccines. 2022;10(1):1–13. doi: 10.3390/vaccines10010091 35062753 PMC8779453

[50] Qiao S, Tam CC, Li X. Risk Exposures, Risk Perceptions, Negative Attitudes Toward General Vaccination, and COVID-19 Vaccine Acceptance Among College Students in south Carolina. Am J Heal Promot. 2022;36(1):175–9. 10.1177/08901171211028407 34164998

[51] Wang Q, Yang L, Jin H, Lin L. Vaccination against COVID-19: A systematic review and meta-analysis of acceptability and its predictors. Prev Med (Baltim) [Internet]. 2021;150(June):106694. Available from: doi: 10.1016/j.ypmed.2021.106694 34171345 PMC8217737

