# Supplementary material for: Determinants of COVID-19 vaccine acceptance in Ethiopia: A systematic review and meta-analysis
Source: PLoS One. 2022 Jun 3;17(6):e0269273. doi: 10.1371/journal.pone.0269273 (PMC9165773; doi:10.1371/journal.pone.0269273)
Supplement: S2 File — (DOCX) [file pone.0269273.s002.docx]

| **Newcastle-Ottawa Quality Assessment Scale scale for cross sectional studies** | **Selection** | | | | **Comparability** | **Outcome** | | **Total score** |
| --- | --- | --- | --- | --- | --- | --- | --- | --- |
|  | Representativeness (1) | Sample size  (1) | Non-respondents  (1) | Ascertainment of the exposure (risk factor)  (2) | The subjects in different outcome groups are comparable, based on the study design or analysis. confounding factors are controlled (2) | Assessment of the outcome  (2) | Statistical test  (1) |  |
| Angelo AT. et al [16] | 1 | 1 | 1 | 1 | 1 | 2 | 1 | 8 |
| Taye BT. et al [25] | 1 | 1 | 1 | 1 | 1 | 1 | 1 | 7 |
| Abebe H. et al [26] | 1 | 1 | 1 | 1 | 1 | 2 | 1 | 8 |
| Zewude B. & Habtegiorgis T.[27] | 1 | 1 | 1 | 1 | 1 | 2 | 1 | 8 |
| Berihun G. et al [28] | 1 | 1 | 1 | 2 | 1 | 2 | 1 | 9 |
| Mose A. [29] | 1 | 1 | 1 | 1 | 1 | 2 | 1 | 8 |
| Shitu K. et al [30] | 1 | 1 | 1 | 2 | 1 | 2 | 1 | 9 |
| Hailemariam S. et al [14] | 1 | 1 | 1 | 2 | 1 | 2 | 1 | 9 |
| Mesfin Y. et al [31] | 1 | 1 | 1 | 1 | 1 | 2 | 1 | 8 |
| Mesele M. [15] | 1 | 1 | 1 | 1 | 1 | 1 | 1 | 7 |
| Mose A. & Yeshaneh A. [32] | 1 | 1 | 1 | 2 | 1 | 2 | 1 | 9 |
| Admasu FT. [33] | 1 | 1 | 1 | 1 | 1 | 2 | 1 | 8 |
